# Highly Efficient Generation of GGTA1 Biallelic Knockout Inbred Mini-Pigs with TALENs

**DOI:** 10.1371/journal.pone.0084250

**Published:** 2013-12-17

**Authors:** Jige Xin, Huaqiang Yang, Nana Fan, Bentian Zhao, Zhen Ouyang, Zhaoming Liu, Yu Zhao, Xiaoping Li, Jun Song, Yi Yang, Qingjian Zou, Quanmei Yan, Yangzhi Zeng, Liangxue Lai

**Affiliations:** 1 Jilin Provincial Key Laboratory of Animal Embryo Engineering, College of Animal Science, Jilin University, Changchun, China; 2 Key Laboratory of Regenerative Biology, Chinese Academy of Sciences, and Guangdong Provincial Key Laboratory of Stem Cells and Regenerative Medicine, South China Institute for Stem Cell Biology and Regenerative Medicine, Guangzhou Institutes of Biomedicine and Health, Guangzhou, China; 3 Key Laboratory of Banna Mini-pig Inbred Line of Yunnan Province, Animal Science and Technology College, Yunnan Agricultural University, Kunming, China; University of Connecticut, United States of America

## Abstract

Inbred mini-pigs are ideal organ donors for future human xenotransplantations because of their clear genetic background, high homozygosity, and high inbreeding endurance. In this study, we chose fibroblast cells from a highly inbred pig line called Banna mini-pig inbred line (BMI) as donor nuclei for nuclear transfer, combining with transcription activator-like effector nucleases (TALENs) and successfully generated α-1,3-galactosyltransferase (GGTA1) gene biallelic knockout (KO) pigs. To validate the efficiency of TALEN vectors, in vitro-transcribed TALEN mRNAs were microinjected into one-cell stage parthenogenetically activated porcine embryos. The efficiency of indel mutations at the GGTA1-targeting loci was as high as 73.1% (19/26) among the parthenogenetic blastocysts. TALENs were co-transfected into porcine fetal fibroblasts of BMI with a plasmid containing neomycin gene. The targeting efficiency reached 89.5% (187/209) among the survived cell clones after a 10 d selection. More remarkably 27.8% (58/209) of colonies were biallelic KO. Five fibroblast cell lines with biallelic KO were chosen as nuclear donors for somatic cell nuclear transfer (SCNT). Three miniature piglets with biallelic mutations of the GGTA1 gene were achieved. Gal epitopes on the surface of cells from all the three biallelic KO piglets were completely absent. The fibroblasts from the GGTA1 null piglets were more resistant to lysis by pooled complement-preserved normal human serum than those from wild-type pigs. These results indicate that a combination of TALENs technology with SCNT can generate biallelic KO pigs directly with high efficiency. The GGTA1 null piglets with inbred features created in this study can provide a new organ source for xenotransplantation research.

## Introduction

Hyperacute rejection (HAR), which is mainly caused by the xenoantigen of galactose-α1,3-galactose (Gal-α1,3Gal), is a major obstacle to pig-to-primate xenotransplantation. Disruption of the α-1,3-galactosyltransferase (GGTA1) gene, which is essential for Gal-α1,3Gal synthesis, is the first step toward overcoming HAR. GGTA1 knockout (KO) swine were generated by several groups through a combination of traditional DNA homologous recombination (HR) and somatic cell nuclear transfer (SCNT) [1,2]. Subsequent studies found that transplantation of hearts from GGTA1 KO pigs to baboons can prolong the graft survival time [3]. Most of the KO pigs previously reported were outbred, except those reported by Lai et al. [1], whose pig population was difficult to expand because of its low fertility. To address this obstacle, we chose the Banna mini-pig inbred line (BMI) with a high fertility in an effort to create a more applicable pig strain for xenotransplantation research.

The Banna mini-pig is a strain of Chinese indigenous pigs with a body weight of less than 50 kg when fully grown. The BMI was established after approximately 30 years of consanguineous inbreeding by a Chinese group. The BMI was developed through more than 20 generations with high inbreeding coefficients [4–6]. BMI is considered as an ideal source for pig to human xenotransplantation to solve the serious shortage of donor organs [7–10]. 

The gene targeting efficiency of traditional DNA HR technology is extremely low. Zinc-finger nucleases (ZFNs) was proven to be a more efficient approach to produce gene KO animals [11–14]. However, the design and assembly of ZFNs require a great deal of optimization to realize specific gene targeting, and ZFNs are unavailable for all target sites [15]. 

Transcription activator-like effector nucleases (TALENs), a new genome-modifying technology, was recently employed for in vivo genetic engineering in vertebrates. Similar to ZFNs, TALENs can mediate DNA double-strand breaks in a specific desired sequence, cause frame-shift mutation, and silence the expression of target genes at high efficiency. TALENs have advantages over ZFNs in many aspects, such as in availability [16], specificity [17], flexibility and lower toxicity [18]. TALENs have been successfully applied for efficient gene targeting in several animal models, including rat [19], zebrafish [20], *Xenopus* [18], mice [21], and rabbit [22]. As of this writing, there are only three reports of KO swine produced with TALENs [16,23,24].

Given the advantages of TALEN technology, we attempted to disrupt the GGTA1 gene in BMI by combining TALEN-mediated gene modification with SCNT. Phenotype analysis and function assay of mutated pigs were also performed. The generation of GGTA1 null BMI pigs provides a more ideal organ source for xenotransplantation research. 

## Results

### Construction of TALENs and Validation of Activity

**Figure 1:**
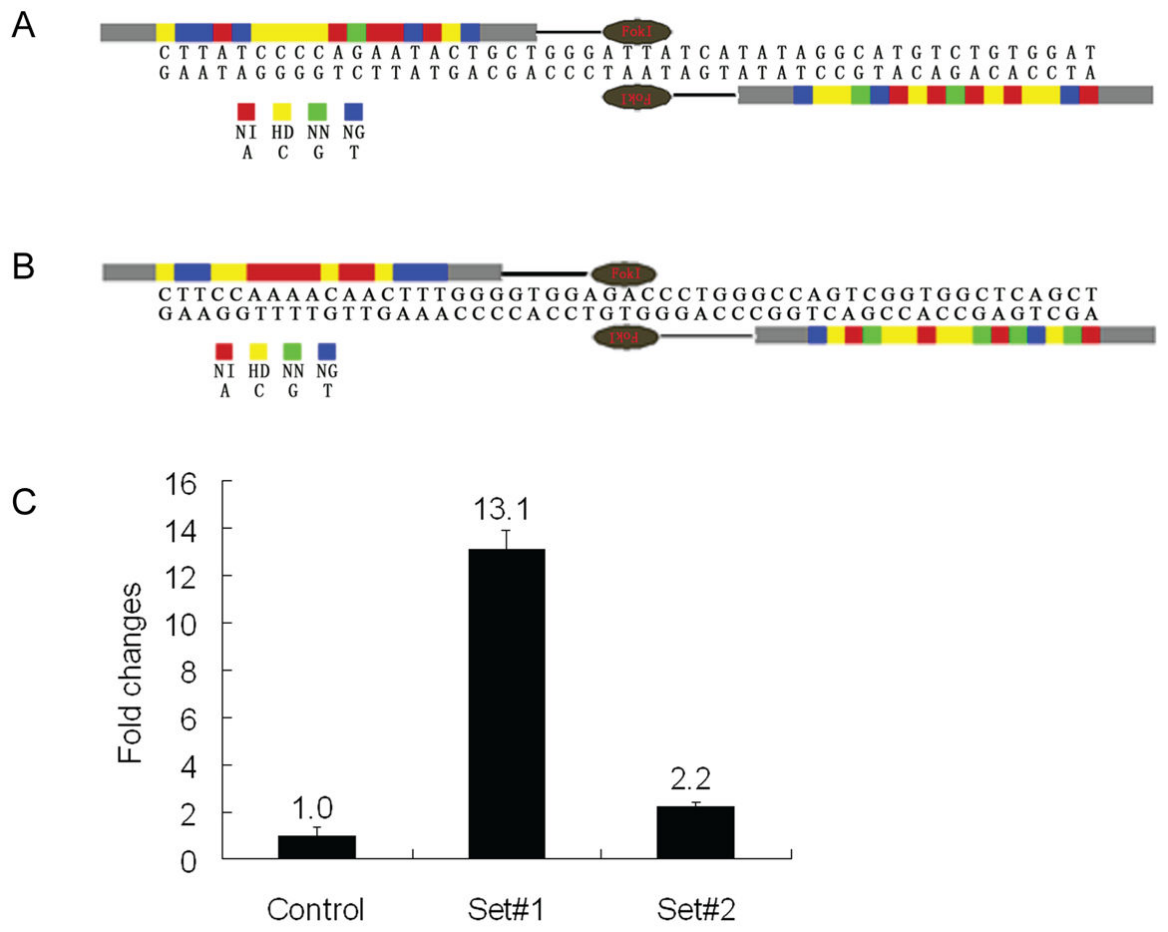
Schematic of TALENs targeting the porcine GGTA1 locus and the activity assay. (A) Schematic of TALEN Set#1. TALEN repeat domains are colored differently to designate the identity of the repeat variable di-residue (RVD). The relationship between RVDs and the cognate-targeted DNA base is NI = A, HD = C, NN = G, NG = T. (B) Schematic of TALEN Set#2. (C) Detection of TALEN activity using luciferase SSA recombination assay. Luciferase activity was increased by 13.1- and 2.2-fold for TALEN Set#1 and Set#2 respectively compared with the control.

Two pairs of TALENs targeting exon 6 of porcine GGTA1 were commercially obtained from ViewSold Biotech. The construction of TALENs are shown in Figures 1A and 1B, respectively. The activity was validated by luciferase single-strand annealing (SSA) recombination assay [20], which showed that TALENs Set#1 had a higher activity over Set#2 (Figure 1C). To evaluate the targeting effectiveness of TALENs in porcine genome, the mRNAs that were in vitro-transcribed from each pair of TALENs were injected into one-cell stage parthenogenetically activated (PA) porcine embryos, and the genotype of the individual embryos were identified in the blastocyst stage as described in our previous research [13]. TALEN Set#1 displayed higher activity, yielding 73.1% (19/26) (Table 1) mutant embryos at 100 ng/μL, whereas TALEN Set#2 had a TALEN-induced mutation ratio of ~7.1%. This result was consistent with that from the luciferase SSA recombination assay. Thus, Set#1 was chosen for the succeeding experiments. We also found that the activity of TALEN Set#1 was dose-dependent in the validation trials using parthenogenetic embryos (73.1% at 100 ng/μL vs. 40.0% at 20 ng/μL vs. 32.0% at 4 ng/μL). The blastocyst rate was not substantially affected with increasing TALEN mRNA concentration (31.3% at 100 ng/μL vs. 35.0% at 20 ng/μL vs. 42.5% at 4 ng/μL).

**Table 1:** Targeting efficiency of TALEN Set#1 in the PA embryo of pigs.

Dose (ng/μL)	Injected	Blastocysts (%)	Screened	Mono-KO (%)^a^	Double KO (%)^b^	Modified (%)^c^
4	80	34(42.5)	25	7(28.0)	1(4.0)	8(32.0)
20	80	28(35.0)	25	4(16.0)	6(24.0)	9(40.0)
100	80	25(31.3)	26	13(50.0)	6(23.1)	19(73.1)
0	50	31(62.0)	4	0	0	0

Three different concentrations of TALEN mRNA were injected into the cytoplasm of PA embryos.

a: Mono-KO/ Screened, b: Double KO/Screened, c: Modified/Screened.

### Generation of GGTA1 KO Porcine Fibroblasts with TALENs

**Figure 2:**
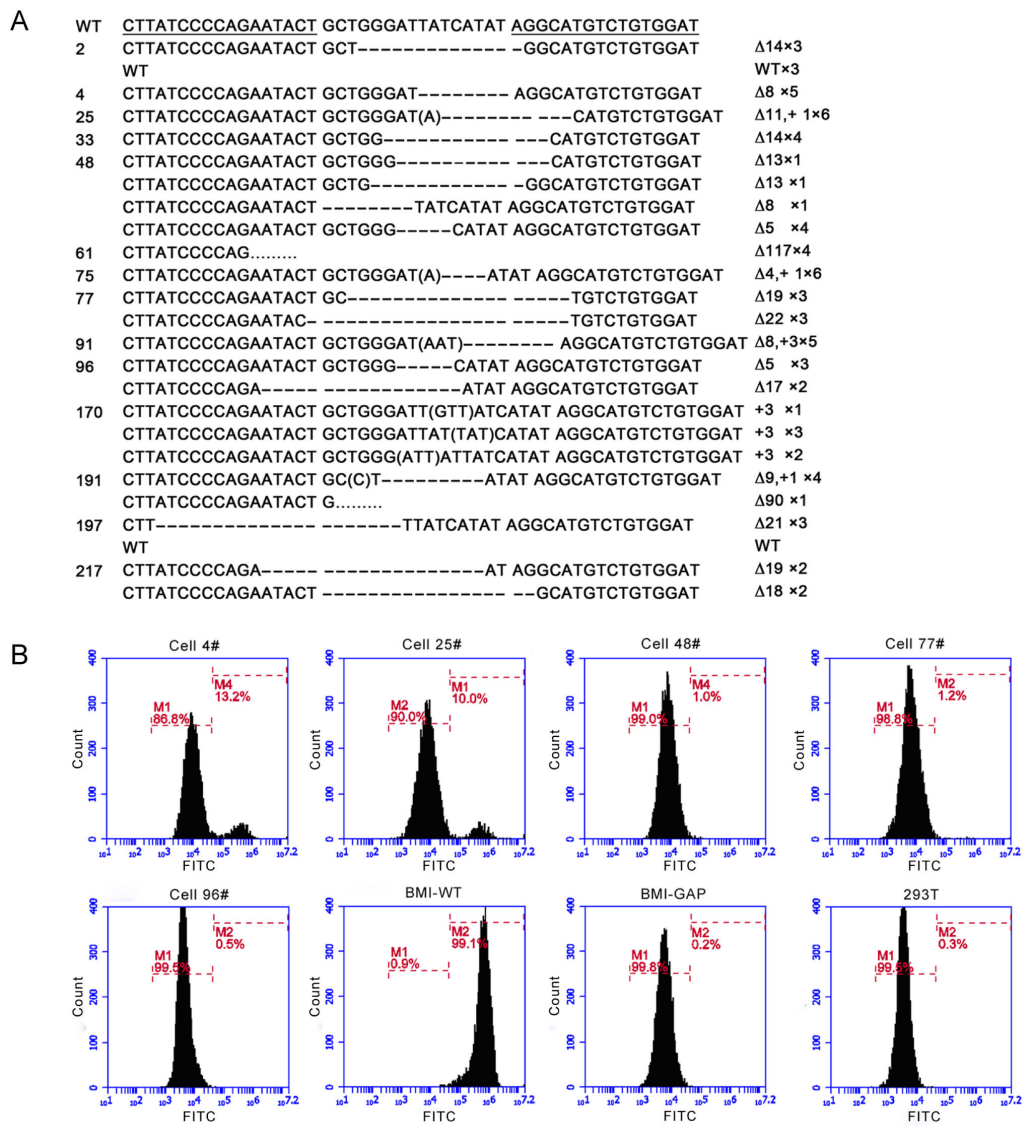
TALEN-mediated mutations in the PFFs, and Gal expression in the mutated PFFs. (A) WT sequence is shown above and TALEN binding sites are underlined. Both deletion and insertion (denoted with “∆” or “+” with the number of base pairs) events are identified. The terms beginning with “×” at the end of each line represent the number of mutations detected by the sequence. (B) Flow cytometric analysis of cells 4, 25,48, 77 and 96 with FITC-conjugated GS-IB4 lectin staining. The horizontal and vertical axes indicate the intensity of fluorescence and the number of events, respectively. The original PFFs of BMI were used as the positive control and 293T cells were as the negative control. The BMI gap was used as the blank control.

Primary porcine fetal fibroblast (PFF) cell line was isolated from a day-35 fetus bred from JS151 strain of BMI. Plasmids of TALEN and pcDNA3.1 vector with a neomycin-resistance gene were co-transfected into BMI fetal fibroblasts by electroporation. Ten days after G418 selection for TALEN Set#1, 209 single cell-derived colonies were picked and screened by PCR DNA sequencing. Among these colonies, 187 cell clones were identified as carrying different mutations in the targeted gene. The frequency of modiﬁcation was 89.5% (187/209) and the rate of biallelic KO was 27.8% (58/209). PCR products were inserted into pMD18-T vector by TA cloning to determine the exact sequence of the mutant (Figure 2A). Among the biallelic KO clones, 37.9% (22/58) of them were homozygous mutations (the same indel in the sister chromatid). However, no mutations were found in all the 56 evaluated cell colonies achieved from TALEN Set#2 plasmid transfection.

The biallelic modified cell colonies (cells 4, 25, 48, 77, 96,) from TALEN Set# 1 were stained with GS-IB4 to detect Gal expression using fluorescence-activated cell sorting (FACS) analysis [1] (Figure 2B). More than 85% of the cells in each of the cell colonies were Gal-α1,3Gal-negative. The remaining positive cells may have been mixed from the non-targeted cells in the process of colony picking.

### Generation of GGTA1 KO Pigs

Six GGTA1 KO cell colonies (cells 4, 25, 33, 77, 96, 219) were used as donor cells for SCNT. A total of 1,919 cloned embryos were transferred into seven estrous surrogates, two of whom became pregnant. One of the surrogates lost pregnancy between 35 d to 42 d after embryo transfer. The other surrogate, who was transferred with cloned embryos derived from cells 25, developed to term and naturally gave birth to three live piglets and a mummy after 124 d of gestation. One pig died soon after birth, whereas the other two were healthy. Sequencing analysis of the genome from all three piglets confirmed a homozygous mutation with an 11-base deletion and one-base insertion (A base) in the target loci, which is consistent with that of cells 25. Cells from the three cloned piglets had normal karyotypes and displayed normal morphology.

### Phenotypes of GGTA1 KO Pigs

**Figure 3:**
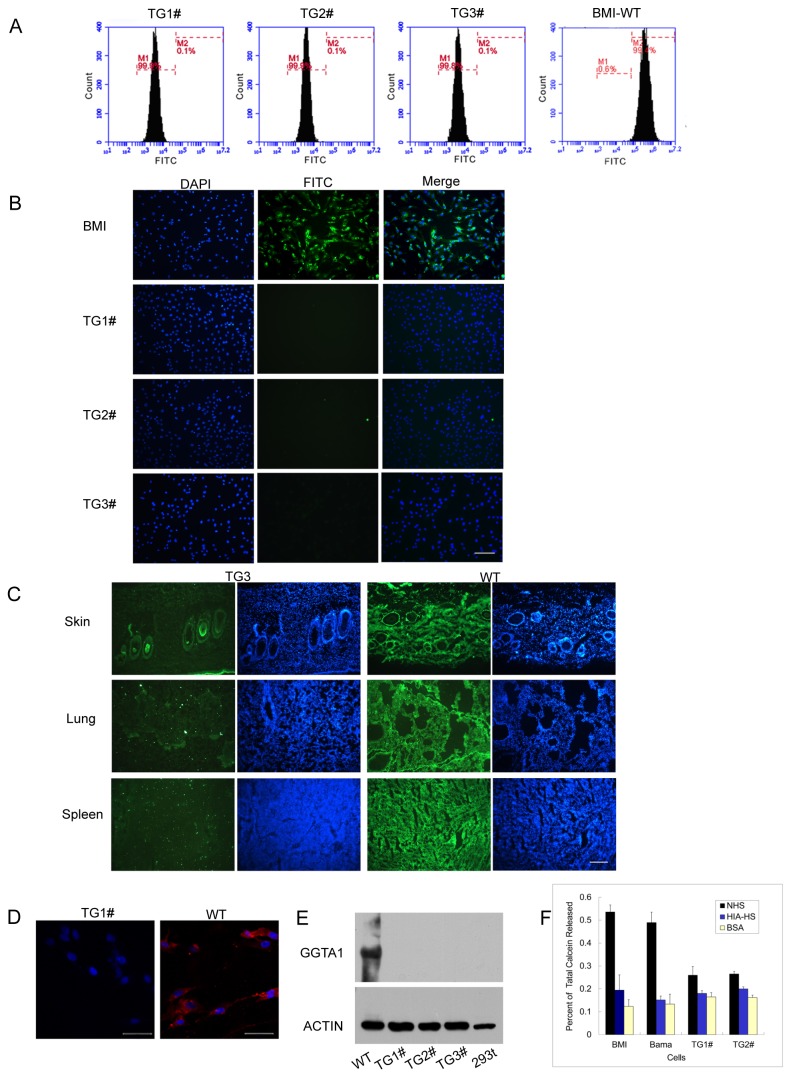
Phenotypes of somatic mutation. (A) Fibroblasts from the cloned pigs were analyzed by FACS with FITC-conjugated GS-IB4 lectin staining. All three pigs were proven to be α 1,3-Gal-negative. (B) Porcine ear fibroblasts stained with FITC-conjugated GS-IB4 were observed under fluorescence microscopy. The original PFFs of BMI were used as the positive control. The scale bar represented 150 μm. (C) The major tissues were cryosectioned and stained with FITC-conjugated GS-IB4. The scale bar represented 250 μm. (D) Immunofluorescence assay. Fibroblasts from cloned pigs were stained with Gal-α1,3Gal-specific mAb M86. The original PFFs of BMI were used as the positive control. The scale bar represents 50 μm. (E) Western blotting for cells of KO piglets and WT pig. β–actin was used as the internal control. (F)Complement-mediated lysis for double KO fibroblasts and WT pig cells. Data shown here are the average of three trials. HIA–HS was used as the negative control. BSA was used as the reagent control.

Ear fibroblasts from the cloned piglets were stained with GS-IB4 lectin and analyzed by FACS. Gal epitopes in the cell surface were completely absent (Figure 3A). The same results were obtained by confocal microscopy (Figure 3B). Histological section analysis with GS-IB4 lectin staining also confirmed that Gal epitope expression could not be discerned from the major organs of piglet TG3#, including the skin lung, spleen(Figure 3C), heart, liver, kidney, intestine, muscle, brain, and stomach(data not shown).

The expression of Gal-α1,3Gal was also evaluated using anti-Gal antibodies (mouse monoclonal Ab (mAb) anti-aGal IgM M86) and subsequent observation by ﬂuorescence microscopy. The ear fibroblasts from the GGTA1-KO piglets did not express Gal-α1,3Gal on their surface (Figure 3D). The western blotting for evaluation GGTA1 expression in the fibroblasts of these cloned piglets, also showed absence of Gal-α1,3Gal(Figure 3E).These results further prove that we obtained homozygous pigs with both GGTA1 alleles inactivated.

The susceptibility of fibroblasts from GGTA1 null piglets to complement-mediated lysis was tested using a previously described protocol [2]. As shown in Figure 3F, only about 25% of the cells from the GGTA1 KO pigs were destroyed by complement-mediated lysis, whereas approximately 50% of the fibroblasts from wild-type (WT) BMI were compromised. This result suggests that fibroblasts from the GGTA1 null piglet were more resistant to lysis caused by pooled complement-preserved normal human serum (NHS) than those from WT BMI and Bama mini-pig (Bama).

### Off-Target Analysis Induced by TALENs

To assay the specificity of TALEN-induced DNA disruption, we employed the protocol described by Yong Lei and Xiaogang Guo [18] in scanning genomic sequences in pigs. A total of 67 potential off-target cleavage sites were predicted (Supplementary information, Table S1). The spacers between the two effector-binding elements (EBEs) in all the potential off-target cleavage sites were greater than 100 bp, which were too long to form Fok I dimerization. Thus, the PCR test that was performed in previous studies to confirm off-target sites is unnecessary [18,22].

## Discussion

GGTA1 is an important gene that accounts for HAR, and GGTA1 KO swine are being increasingly used to promote organ transplantation from pigs to primates. Compared with previous similar efforts, this study has two unique aspects. The first one is that a highly inbred pig line of BMI was used as the donor for SCNT. With the inbred pigs, once the genetically modified animals were cloned, the population with the same genetic modification could be expanded easily through consanguineous mating without severe depression. Thus, reliable and stable transgenic animal strains could be established.

The second aspect of this study is that a newly emerging gene disruption technology, TALENs, was employed for genetic modification. TALEN DNA or encoding mRNA to embryos were injected to generate targeted mutations in small animals, including rat [19], zebraﬁsh [20], *Xenopus* [18], mice [21], and rabbit [22]. However, many of the resulted founder animals were chimeric ones with multiple mutations through the embryo injection [22]. To get the animals with a single mutation, one or two more round of further breeding have to be employed for selection among the offspring. The injection of TALENs into embryos is not so applicable in large animals as in small animals, because large animals, such as pigs, have long gestation cycles, high recipient costs, which make it difficult to achieve large animal offspring with a single expected mutation. Therefore, in this study, we chose gene targeting in somatic cells using TALEN technology followed by SCNT to produce GGTA1 KO pigs. 

To validate TALEN activity in pig genomics, we microinjected TALEN-coding mRNA into PA embryos and identified the genotype of the individual embryos at the blastocyst stage. We found that the efficiency of TALEN Set#1 was higher than that of Set#2, which was consistent with the results of luciferase SSA recombination assay. We also observed a dose-dependent targeting efficiency in the TALEN Set#1 mRNAs. The functional concentration of TALENs was over a very wide dosage range (4 ng/μL to 100 ng/μL), and the embryos were more tolerant at high doses than ZFNs [16,18]. The early in vitro development of the embryos did not appear to be substantially affected by the injection of different TALEN concentrations.

Given that somatic cell modification followed by cloning is more appropriate for the generation of transgenic pigs, we transfected primary fetal ﬁbroblasts with TALENs by co-transfection. The gene targeting efficiency in PFFs was 89.5%, which was significantly higher than using the traditional methods. Even more notably, we were able to get the biallelic KO fibroblasts, which were impossible by using traditional HR method, and the rate was up to 27.8%. Various mutants were observed at the targeted loci, including small or vast deletions (e.g., -1 base or -117 base), a few insertions (e.g., +3 base), or two cases presenting both (e.g. +1 base or -11 base). More deletion mutants than insertion mutants were observed in our study, which was consistent with the observation in a report on microinjection of mice embryos[21]. Among biallelic modification colonies, high portions (22 of 58) were homozygous for the same indel. Similar ﬁndings have also been previously reported. It could be caused by sister chromatid exchange as suggested by Carlson DF et al [16].

We have successfully produced live piglets by using GGTA1 biallelic KO cells as donor nuclei for SCNT. Genotype assay by PCR sequencing confirmed that the mutation pattern in the cloned piglets was consistent with that in the donor cells. The mutation of the GGTA1 gene led to a complete loss of its function in the cloned pigs, which was confirmed by GS-IB4 lectin staining and anti-Gal antibody treatment, followed by FACS analysis, ﬂuorescence microscopy or western blot assay. The fibroblasts from the GGTA1 null piglets were more resistant to lysis by pooled complement-preserved NHS than those from WT pigs, which suggests that the organs and tissues in these pigs can overcome HAR upon transplantation into primates.

Thus, we efficiently generated GGTA1 KO BMI pigs with TALENs. The results in our study further confirm that TALENs can be used conveniently and efficiently for specific gene modification in large animals. The GGTA1 null piglets with inbred features produced in this study provide a new organ source for xenotransplantation research.

## Materials and Methods

### Ethical Approval

The animal experiments were approved by the Institutional Animal Care and Use Committee of Guangzhou Institute of Biomedicine and Health, Chinese Academy of Sciences(ID 2012040) and the Department of Science and Technology of Guangdong Province (ID SYXK 2005-0063). And it complied with the guidelines of Institutional Animal Care and Use Committee of Guangzhou Institute of Biomedicine and Health, Chinese Academy of Sciences (Animal Welfare Assurance #A5748-01). All surgical procedures were performed under anesthesia, and all efforts were made to minimize animal suffering.

### TALEN Design and Generation

TALENs were designed to target exon 6 of the porcine GGTA1 gene. The assembly and validation of TALENs were completed by ViewSold Biotech according to Golden Gate TALEN assembly. SSA recombination assay was employed to evaluate the targeting efficiency of TALEN vector in vitro and the detail method was described in a previous study [20]. Briefly, 293T cells in 24-well plates were transfected with 200ng of TALENs expression plasmids, 50ng SSA reporter plasmid and 10ng Renilla plasmid. Each experiment was triplicated. The cells were harvested 1d after transfection and treated with Luciferase Cell Lysis Buffer (NEB). The relative luciferase activity was detected.

### PA Embryo Production

Methods used for porcine oocyte collection, in vitro maturation, and PA were similar to our previous studies [13,25,26]. Pig ovaries were collected from a local slaughterhouse and transported to the laboratory in 0.9% saline between 35 °C and 39 °C. Cumulus oocyte complexes (COCs) were aspirated from antral follicles with 2 mm to 6 mm diameter. The COCs were washed with the maturation medium, Tissue Culture Medium 199 (Gibco), which was supplemented with 0.1% (w/v) polyvinyl alcohol (Sigma), 3.05 mM D-glucose, 0.91 mM sodium pyruvate (Sigma), 0.57 mM cysteine (Sigma), 0.5 mg/mL luteinizing hormone (LH Sigma), 0.5 mg/mL follicle stimulating hormone (FSH Sigma), 10 ng/mL epidermal growth factor (Sigma), 10% (v/v) porcine follicular fluid, 75 mg/mL penicillin G, and 50 mg/mL streptomycin. The COCs were transferred to a four-well multidish (Nunc) containing 500 mL of the maturation medium. The multidish was previously covered with mineral oil and pre-equilibrated at 39 °C in an atmosphere of 5% CO_2_ for more than 3 h. After 42 h to 44 h of culture maturation, the oocytes were released from the cumulus cells by vigorous vortexing for 5 min in TL–HEPES containing 0.1% hyaluronidase (Sigma). Cumulus-free oocytes were placed between two 0.2 mm diameter platinum electrodes (1 mm apart) in a fusion and activation medium (0.3 M mannitol, 1 mM CaCl_2_·2H_2_O, 0.1 mM MgCl_2_·6H_2_O, and 0.5 mM HEPES). Activation was induced by two successive direct current(DC) pulses of 1.2 kV/cm for 30 μs on an electrofusion instrument (CF-150B, BLS, Hungary).

### TALEN mRNA Cytoplasm Microinjection

TALEN mRNA was transcribed in vitro and polyadenylated using mMESSAGE kit, mMACHINE SP6 (Ambion), and poly(A) polymerase (Takara). TALEN mRNAs were resuspended in RNAse-free water, and their concentrations were measured. The solutions were diluted at 4, 20, and 100 ng/μL in 5 μL aliquots. The TALEN mRNA solutions were stored at –80 °C and kept on ice during the microinjection process.

One-cell stage porcine PA embryos were used for mRNA injection, and approximately 1 pL to 2 pL of mRNA was injected into the cytoplasm of embryos using a glass pipette (1 μm to 3 μm diameter) and a micromanipulator. The injected embryos were washed thrice with embryo culture medium (PZM-3) and transferred into 500 μL of culture medium in a four-well multidish covered with mineral oil. The embryos were incubated at 39 °C in a 5% CO_2_ atmosphere for 6 d. The blastocyst rate was counted, and the blastocysts were selected for genotyping.

### Cell culture, Transfection, and Selection

PFFs were isolated from 35-day-old Chinese BMI fetuses and digested by collagenase IV-DNase in cell culture medium containing 0.32 mg/mL collagenase IV and 2500 IU/mL DNase for 4 h to 6 h at 39 °C. PFFs were cultured in 10 cm dishes for 12 h and frozen in fetal bovine serum (FBS) containing 10% dimethylsulfoxide for future use. A day before the transfection, PFFs were thawed and cultured in 10 cm dishes until subconfluent. Approximately 1 × 10^7^ PFFs in 600 μL D-PBS containing 50 μg of the TALEN plasmid pair and 50 μg of the linearized pcDNA3.1 were electroporated at 230 V and 500 μF using a Gene Pulser Xcell electroporator (Bio-Rad). The transfected cells were divided into twenty 10 cm diameter dishes and recovered for 2 d in Dulbecco’s modified Eagle’s medium containing 15% FBS. The cells were selected with 800 μg/mL G418 (Merck) for 8 d to 10 d. Cell colonies were picked and cultured in 48-well plates. After 2 d, the cell colonies were subcultured and a fraction was selected for PCR genotyping. Then, the cells were frozen for future use.

### SCNT and Gene KO Pig Generation

Maturate oocytes were enucleated by aspirating the first polar body and adjacent cytoplasm with a glass pipette in a manipulation medium of HEPES-buffered M199 plus cytochalasin B (7.5 μg/mL). The cells identified as biallelic KO by gene sequencing were thawed and used as donor cells to be injected into the perivitelline space of the oocytes. Fusion and activation were performed with two successive DC pulses at 1.2 kV/cm for 30 μs using an electrofusion instrument. The reconstructed embryos were cultured in PZM3 at 39 °C overnight and surgically transferred to the oviducts of the surrogates that were observed in estrus the day before. The pregnancy status was monitored by ultrasonography 24 d after embryo transfer. Once detected, the pregnancy status was monitored weekly until delivery. The cloned piglets were delivered by natural birth. The genomic DNA that was isolated from the ear skin biopsy of the newborn cloned piglets was used for PCR genotyping, and the fibroblasts were isolated and cultured by methods similar to PFFs.

### Genotype Identification

The injected PA embryos that were developing into blastocyst stage were lysed individually in 6 μL of NP-40 solution (0.45% NP-40 plus 0.6% Proteinase K) for 25 min at 56 °C and 4 min at 90 °C. Then, they were used as templates for PCR. The primers were designed to amplify across the target sites. The TALEN Set#1 forward (5’-CATCACTCAGGAGTGCTTCAA-3’) and reverse (5’-ACAAGGCTCAAAGTTGCAAG-3’) primers yielded a 384 bp amplification product, and the TALEN Set#2 forward (5’-CTGGGTCCTCTGCGTTCCT-3’) and reverse (5’-GAGTGATGTTTAGAACCTGAGTGGG-3’) primers yielded a 355 bp amplification product. The PCR products were recovered using an agarose gel DNA purification kit (Takara) and sequenced (BGI, Shenzhen, China) to identify the mutation. The genomic DNA from WT PA embryos was used as the negative control. 

Genotype identification for cell clones was similar to that for the embryos described above. A small fraction of cells from a single cell clone was lysed individually in 10 μL of NP-40 solution for 90 min at 56 °C and 10 min at 95 °C. The PCR conditions were the same as above, and the PCR products were recovered and sequenced to identify the mutants. Some PCR products were selected to clone into a pMD-18T plasmid vector (Takara) and sequenced to determine the exact mutant sequences.

Genomic DNA was extracted from the ear tissues of each newborn cloned piglet using a TIANamp Genomic DNA kit (Tiangen). The genome was assessed for mutagenesis at the TALEN-targeted site by PCR-based assays to detect the cell mutations.

### Flow Cytometric Analysis

Pig cells were stained with FITC-GS-IB4 lectin (Sigma) to analyze Gal-α1,3Gal epitope expression. The harvested cells were washed thrice with PBS and stained for 5 min at 37 °C in 20 μg/mL lectin. The cells were washed, resuspended with 300 μL of PBS, and analyzed using a BD Accuri C6 flow cytometer.

### Fluorescent Microscopy

Fibroblasts were cultured on coverslips for 24 h, fixed with 4% paraformaldehyde for 10 min, and washed with PBS with 2 mg/mL glycin. Cells were incubated with 0.2% Triton X-100 (Sigma) for 10 min at room temperature and washed. Various tissues of a dead clone pig, including the heart, liver, spleen, lung, kidney, skin, intestine, and muscle, were obtained. Frozen sections were cut to 5 µm thickness, fixed briefly in 100% methanol for 10 min, and washed with PBS thrice for 5 min each. The fibroblasts and tissues were blocked with 1% bovine serum albumin (BSA) in PBS for 1 h at room temperature and incubated overnight in a humid chamber at 4 °C with 40 µg/mL FITC–GS–IB4 in blocking buffer. The slides were washed with PBS, and the nuclei were counterstained with 1 µg/mL DAPI. The slides were covered with mounting medium and observed using fluorescence microscopy (Olympus BX51) under the appropriate excitation filters.

### Immunofluorescence Assay

Both WT and GalT KO fibroblasts were grown in 24-well plates. After 24 h of culture, both the WT and GalT KO fibroblast monolayers were ﬁxed with 4% paraformaldehyde for 10 min. The monolayers were blocked with 1% BSA in PBS for 1 h and incubated with mAb anti-aGal IgM M86 (Alexis) overnight in a humid chamber at 4 °C. The cells were washed thrice with PBS and incubated for 2 h with a secondary antibody (goat anti-mouse IgM, Bioss). The nuclei were counterstained with DAPI. Coverslips were mounted on glass slides, and the slides were analyzed by ﬂuorescence microscopy (Olympus).

### Western blot Assay

Ear fibroblasts isolated from these piglets were lysed in RIPA buffer (9806, Cell Signaling, Danvers, MA, USA) containing 1%(v/v) Protease Inhibitor Cocktail (P8340, Sigma, St. Louis, MO, USA), diluted in 1× SDS sample buffer (62.6 mM Tris-HCl, pH6.8, 2%SDS, 10%glycerol, and 0.01% bromophenol blue) and incubated at 100 °C for 5 min. The proteins in the cell lysates were separated via SDS-PAGE and transferred electrophoretically onto a polyvinylidene difluoride membrane (PVDF; Millipore, Bedford, MA, USA). The membranes were incubated overnight with the mAb (M86) (1:10, ALX-801-090-1, Enzo, Lausen, Switzerland) at 4 °C followed by incubation with peroxidase conjugated goat anti-mouse IgM secondary antibody (bs-0368G-HRP, Boiss, China) for 1 h at room temperature. The GGTA1 proteins were detected using a SuperSignal West PicoTrial enhanced chemiluminescence kit (Thermo, Rockford, IL, USA). β-actin was probed as a loading control. Fibroblasts from age-matched wild-type Banna pig were used as positive control and 293t cells were the negative control.

### Complement Lysis Assay

A complement lysis assay on the fibroblasts of three cloned pigs was performed using WT PFFs of BMI as the control. The process was described in detail in a previous study [2]. In brief, cells of 80% confluence in 24-well plates were incubated with 250 µL of 2 µg/mL calcein AM (Molecular Probes) for 1 h at 37 °C and washed with PBS. Cells were mixed with 250 µL of 20% NHS (Innovative), 20% heat-inactivated human serum (HIA–HS), and 0.5% BSA diluted in gelatin veronal buffer (Alfa Aesar). Then, cells were incubated for 1 h at 37 °C. Cell supernatants in wells were transferred into a 96-well plate (sample 1), and 250 µL of 0.1% Triton X-100 (Sigma) was added to each well to lyse the remaining cells for 15 min at room temperature. Then, the lysed cells were moved to a new 96-well plate (sample 2). Cell lysis was determined by fluorescein detection using a microplate detector (PE, Victor 3) at 485 nm/535 nm. Detection was repeated thrice in this experiment. The data were averaged using the following formula: % Calcein release = (Sample 1)/(Sample 1 + Sample 2) × 100%.

### Off-target Analysis

TALEN off-target cleavage sites were predicted by electronic-PCR (www.ncbi.nlm.nih.gov/sutils/e-pcr), and potential off-target sites were searched as previously described [18,22]. In brief, the sequences, which conformed with the criteria of having up to six mismatches, 2 bp gaps between two EBEs, and < 1,000 bp between the two putative off-target sites, were picked up. These regions of potential off-target sites were amplified in biallelic KO animals and tested for cleavage using PCR sequencing. However, if the spacers between EBEs in the potential off-target cleavage sites were greater than 100 bp, the PCR test for confirmation of off-target sites is unnecessary.

## Supporting Information

Table S1 **Potential off-target sites of the TALENs Set#1 identified by e-PCR in the porcin genome.** In total, 67 potential off-target sites were predicted under the criteria of having up to six mismatches, 2 bp gaps between two EBEs, and < 1,000 bp between the two putative off-target sites. Among these, no sites had a spacers of < 100 bp between the two binding sequences.
(DOC)
