# Supplementary material for: Highly Efficient Generation of GGTA1 Biallelic Knockout Inbred Mini-Pigs with TALENs
Source: PLoS One. 2013 Dec 17;8(12):e84250. doi: 10.1371/journal.pone.0084250 (PMC3866186; doi:10.1371/journal.pone.0084250)
Supplement: Table S1 — Potential off-target sites of the TALENs Set#1 identified by e-PCR in the porcin genome. In total, 67 potential off-target sites were predicted under the criteria of having up to six mismatches, 2 bp gaps between two EBEs, and < 1,000 bp between the two putative off-target sites. Among these, no sites had a spacers of < 100 bp between the two binding sequences. (DOC) [file pone.0084250.s001.doc]

Table S1 Potential off-target sites of the TALENs used here identified by e-PCR in the porcin genome

| chr | strand | strand from to | mism | gaps | act_len/exp_len |
| --- | --- | --- | --- | --- | --- |
| 1 | + | 152718846-152719565 | 4 | 2 | 720/0-1000 |
| 1 | + | 146065481-146065731 | 6 | 2 | 251/0-1000 |
| 1 | + | 134511070-134511716 | 5 | 1 | 647/0-1000 |
| 1 | - | 134337004-134337814 | 6 | 1 | 811/0-1000 |
| 1 | - | 107866968-107867545 | 6 | 2 | 578/0-1000 |
| 1 | - | 81344380-81345124 | 6 | 1 | 745/0-1000 |
| 1 | - | 58132462-58133235 | 5 | 2 | 774/0-1000 |
| 1 | - | 21286311-21287030 | 5 | 2 | 720/0-1000 |
| 1 | + | 98691972-98692571 | 5 | 2 | 600/0-1000 |
| 1 | - | 143637638-143638383 | 6 | 2 | 746/0-1000 |
| 1^a^ | - | 136616703-136616751 | 0 | 0 | 49/0-1000 |
| 1 | - | 52900965-52901406 | 6 | 2 | 442/0-1000 |
| 1 | - | 6914530-6915007 | 5 | 2 | 478/0-1000 |
| 2 | + | 96425064-96425477 | 6 | 1 | 414/0-1000 |
| 2 | + | 80914269-80914677 | 5 | 1 | 409/0-1000 |
| 2 | + | 4619147-4619833 | 6 | 2 | 687/0-1000 |
| 3 | - | 140160280-140160994 | 6 | 2 | 715/0-1000 |
| 3 | - | 136038398-136038709 | 6 | 1 | 312/0-1000 |
| 3 | - | 55717333-55717437 | 6 | 2 | 105/0-1000 |
| 3 | - | 35343792-35344391 | 6 | 1 | 600/0-1000 |
| 3 | - | 15569937-15570849 | 6 | 2 | 913/0-1000 |
| 3 | - | 10848235-10848616 | 6 | 2 | 382/0-1000 |
| 4 | + | 107052157-107052628 | 5 | 1 | 472/0-1000 |
| 4 | + | 43419934-43420116 | 6 | 2 | 183/0-1000 |
| 4 | - | 107319822-107320596 | 6 | 2 | 775/0-1000 |
| 4 | - | 87810387-87811076 | 6 | 2 | 690/0-1000 |
| 4 | - | 74876087-74876739 | 6 | 2 | 653/0-1000 |
| 5 | + | 67069326-67069616 | 6 | 2 | 291/0-1000 |
| 5 | - | 106283696-106284118 | 4 | 2 | 423/0-1000 |
| 5 | - | 97565867-97566627 | 4 | 2 | 761/0-1000 |
| 6 | + | 143899352-143900022 | 6 | 0 | 671/0-1000 |
| 6 | + | 89671941-89672641 | 6 | 2 | 701/0-1000 |
| 6 | + | 11513323-11513580 | 4 | 1 | 258/0-1000 |
| 6 | - | 48444955-48445280 | 6 | 2 | 326/0-1000 |
| 6 | - | 1955320-1955499 | 6 | 2 | 180/0-1000 |
| 7 | + | 57967575-57968041 | 6 | 2 | 467/0-1000 |
| 7 | - | 125298914-125299215 | 6 | 2 | 302/0-1000 |
| 7 | - | 49573915-49574487 | 6 | 2 | 573/0-1000 |
| 7 | - | 22414477-22415388 | 4 | 2 | 912/0-1000 |
| 8 | + | 80898434-80899009 | 5 | 2 | 576/0-1000 |
| 8 | + | 32843939-32844484 | 6 | 1 | 546/0-1000 |
| 8 | - | 145069226-145070055 | 4 | 2 | 830/0-1000 |
| 8 | - | 44525187-44525287 | 6 | 2 | 101/0-1000 |
| 9 | - | 118245154-118245606 | 5 | 1 | 453/0-1000 |
| 9 | - | 88006522-88007541 | 5 | 1 | 1020/0-1000 |
| 9 | - | 33591681-33592216 | 6 | 2 | 536/0-1000 |
| 9 | - | 124859-125154 | 6 | 2 | 296/0-1000 |
| 10 | + | 70739251-70739637 | 6 | 2 | 387/0-1000 |
| 10 | + | 8364163-8364534 | 5 | 2 | 372/0-1000 |
| 10 | - | 36389081-36390115 | 6 | 2 | 1035/0-1000 |
| 11 | + | 27075457-27076058 | 6 | 2 | 602/0-1000 |
| 12 | - | 39721348-39721923 | 5 | 2 | 576/0-1000 |
| 13 | + | 41185786-41185963 | 6 | 2 | 178/0-1000 |
| 13 | + | 32607146-32607446 | 6 | 2 | 301/0-1000 |
| 13 | + | 30504106-30504749 | 6 | 2 | 644/0-1000 |
| 13 | - | 41048803-41048980 | 6 | 2 | 178/0-1000 |
| 14 | + | 111743360-111744204 | 6 | 2 | 845/0-1000 |
| 14 | - | 117263501-117263777 | 6 | 1 | 277/0-1000 |
| 14 | - | 112969862-112970364 | 6 | 2 | 503/0-1000 |
| 14 | - | 76272003-76272534 | 5 | 1 | 532/0-1000 |
| 15 | + | 123526377-123526680 | 6 | 1 | 304/0-1000 |
| 16 | - | 46227756-46228090 | 6 | 2 | 335/0-1000 |
| 16 | - | 26930223-26930565 | 6 | 2 | 343/0-1000 |
| 17 | - | 36208061-36208370 | 6 | 2 | 310/0-1000 |
| 18 | - | 101326-102368 | 6 | 2 | 1043/0-1000 |
| X | + | 97688085-97688354 | 5 | 2 | 270/0-1000 |
| X | - | 114346063-114347042 | 5 | 2 | 980/0-1000 |

a: The hits in red are the EBEs used in this study.
